# Persistent Pulmonary Hypertension of the Newborn: A Pragmatic Review of Pathophysiology, Diagnosis, and Advances in Management

**DOI:** 10.3390/biomedicines13102332

**Published:** 2025-09-24

**Authors:** Karolina Chojnacka, Yogen Singh, Sheen Gahlaut, Witold Blaz, Agata Jerzak, Tomasz Szczapa

**Affiliations:** 1 II Department of Neonatology, Neonatal Biophisical Monitoring and Cardiopulmonary Therapies Research Unit, Poznan University of Medical Sciences, 60-535 Poznan, Poland; agata.jerzak@gmail.com (A.J.); tszczapa@gmail.com (T.S.); 2 Department of Pediatrics, Division of Neonatology, University of California, UC Davis Children’s Hospital, Sacramento, CA 95817, USA; ygsingh@health.ucdavis.edu; 3 Medical Sciences Division, Lincoln College, Oxford University, Oxford OX1 2JD, UK; sheen.gahlaut@lincoln.ox.ac.uk; 4 Provincial Hospital No 2 Saint Jadwiga the Queen Clinical Provincial Hospital, University of Rzeszow, 35-301 Rzeszow, Poland; witekblaz@yahoo.com

**Keywords:** neonatal diseases, pathophysiology, innovative therapeutic approaches, persistent pulmonary hypertension

## Abstract

Persistent pulmonary hypertension of the newborn (PPHN) results from disrupted fetal–neonatal circulatory transition, characterized by elevated pulmonary vascular resistance (PVR), right-to-left shunting, and refractory hypoxemia. Despite improved perinatal care, PPHN remains a major source of neonatal morbidity and mortality. This review details PPHN phenotypes, pathophysiology, etiology, diagnostics including echocardiography and biomarkers like B-type Natriuretic Peptide (BNP) or N-terminal pro-B-type Natriuretic Peptide (NT-proBNP), and current therapeutic modalities, from lung recruitment and surfactant to targeted vasodilator therapy (iNO, sildenafil, milrinone, bosentan) and extracorporeal membrane oxygenation (ECMO). We emphasize the role of endothelial and molecular mechanisms in precision therapy and outline guidelines for clinical decision-making in diverse care settings.

## 1. Introduction

Persistent pulmonary hypertension of the newborn (PPHN) is a condition resulting from a disrupted transition from fetal to neonatal circulation. It is characterized by sustained elevation of pulmonary vascular resistance (PVR) which leads to right-to-left or bi-directional shunting of blood through the foramen ovale and ductus arteriosus, and it prevents an increase in pulmonary blood flow (PBF). Therefore, newborns with PPHN suffer from persistent severe hypoxemia; this clinically presents as respiratory distress shortly after birth and often requires immediate intervention. Despite advances in perinatal care, PPHN remains a significant cause of morbidity and mortality in newborns [1,2].

**Figure 1:**
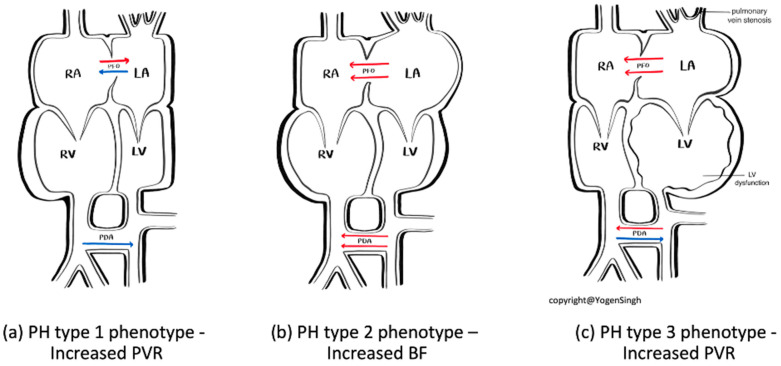
Phenotypes of pulmonary hypertension: Type 1—primarily increased pulmonary vascular resistance (most common type); Type 2—increased pulmonary blood flow leading to pulmonary hypertension; and Type 3—increased pulmonary wedge pressure from increased pulmonary venous pressure (least common type, such as in pulmonary vein stenosis and left ventricle dysfunction).

The etiology of PPHN is multifactorial, and the process underlying the development of high pulmonary resistance is complex and still not fully understood. There are a range of different phenotypes of PPHN which can lead to acute pulmonary hypertension: (1) raised PVR at pre-capillary level, which is the most common phenotype; (2) increased pulmonary blood flow and pulmonary vascular remodeling (in conditions such as left-to-right shunt lesions and arterio-venous malformations); and (3) increased pulmonary venous hypertension at the post-capillary level such as in obstructed total anomalous pulmonary venous connections or severe left ventricular disfunction (Figure 1). It is crucial to understand the phenotype and underlying pathophysiology, as these can result in different management strategies. In the past decade, significant progress has been made towards developing new therapies. There is also a growing need to develop standardized guidelines that can help clinicians in decision-making when treating patients with this inhomogeneous condition [3]. Optimizing cardiorespiratory management is the most common approach; this includes lung recruitment to optimize hypoxia treatment, surfactant therapy, conventional or high-frequency ventilation, and pulmonary vasodilators such as nitric oxide, milrinone, or sildenafil. However, despite optimizing cardiovascular support with conventional therapy (including HFOV, iNO, additional vasodilator therapy, and cardioactive medications), in some cases, extracorporeal membrane oxygenation (ECMO) is needed. A better understanding of the processes occurring in pulmonary endothelial and smooth muscle cells can help in selecting the appropriate therapy for a specific patient, as frequently employed traditional approaches may not suffice [2].

Beyond its immediate respiratory implications, PPHN poses additional risks to affected infants. They are more susceptible to complications such as neurological impairment, cardiovascular dysfunction, bronchopulmonary dysplasia (BPD), and long-term developmental delays [4].

The impact of PPHN on newborn health reverberates through the neonatal period and beyond, underscoring the importance of early detection, prompt intervention, and comprehensive follow-up care. This article endeavors to provide a pragmatic approach towards the management of PPHN in clinical practice, encompassing its pathophysiology, etiology, diagnostic tools, and therapeutic approaches [5].

## 2. Search Strategy and Selection Criteria

This narrative review on persistent pulmonary hypertension of the newborn (PPHN) is based on studies identified through PubMed/MEDLINE, Embase, and the Cochrane Library (1997–2025) to capture both cornerstone historical studies (e.g., early nitric oxide trials) and contemporary advances. Search terms included PPHN, pulmonary vasodilators, inhaled nitric oxide, ECMO, biomarkers, and neonatal pulmonary hypertension. We included RCTs, observational studies, systematic reviews, experimental work, and expert statements focused on neonates. Non-neonatal and non-English publications were excluded.

## 3. Etiology

The etiology of PPHN is multifactorial, involving a combination of genetic, environmental, and physiological factors [6]. Initially understood as a failure of the neonatal pulmonary circulation to transition from fetal circulation (which results in sustained high vascular resistance), the understanding of PPHN has significantly evolved.

Research in the 1980s identified the critical role of the ductus arteriosus in maintaining high PVR, pivotal for understanding the pathophysiology of PPHN [6]. PPHN can occur from primary pulmonary pathology or secondary conditions such as meconium aspiration, infections, or congenital anomalies like diaphragmatic hernia [6]. These conditions may prevent the normal postnatal decrease in PVR, leading to severe respiratory distress. Contributing factors include fetal hypoxia, acidosis, and structural immaturity of the lungs [6]. Additionally, environmental influences during pregnancy such as maternal medication use or inflammation, along with genetic predispositions, are significant in the development of PPHN.

Secondary PPHN is often linked to conditions affecting the pulmonary or cardiovascular systems of the newborn, including respiratory distress syndrome, pneumonia, and conditions like sepsis or asphyxia [6]. Interactions between genetic predispositions and environmental factors during pregnancy, such as maternal smoking or diabetes, can significantly increase the risk of developing PPHN. These factors may induce hypoxia and inflammation, leading to epigenetic modifications that affect gene expression related to pulmonary vascular tone and reactivity [7].

The introduction of inhaled nitric oxide (iNO) in the 1990s revolutionized treatment, significantly reducing pulmonary vascular resistance and enhancing oxygenation in affected neonates [8,9,10,11]. Contemporary studies have expanded to include the roles of endothelin-1, prostacyclin, and detailed genetic and molecular foundations of PPHN [8]. Such research is refining treatment strategies and exploring new pharmacological interventions, aiming to improve outcomes further.

The underlying mechanisms involve an intricate interplay between vasoactive mediators, endothelial dysfunction, and mechanical factors, including an impaired response to oxygen and vasoactive stimuli like endothelin and nitric oxide [8]. These mediators are essential for regulating vascular tone. Moreover, specific genetic variants, such as polymorphisms in the gene encoding endothelial nitric oxide synthase (eNOS), have been linked to PPHN. Deficiencies or dysfunctions in eNOS can lead to inadequate nitric oxide production, which is a crucial factor in reducing pulmonary vascular resistance immediately after birth [8,9,10].

As research continues to delve into these complex interactions, our understanding improves, guiding more effective and targeted therapeutic approaches. This comprehensive exploration of PPHN’s causes is essential for developing novel interventions that can better manage or even prevent this challenging condition in neonates.

## 4. Clinical Presentation and Diagnosis

Clinically, PPHN presents with severe hypoxemia and cyanosis, which result from the under-oxygenation of arterial blood. Infants with PPHN also often show signs of respiratory distress, leading to respiratory failure. Heart auscultation may reveal flow murmurs due to excessive blood flow through the lungs, from PDA shunt, tricuspid regurgitation or sometimes from the associated underlying congenital heart defect. Additionally, these infants typically demonstrate a poor response to oxygen supplementation, where increasing oxygen delivery does not adequately improve blood oxygen levels. This condition requires immediate medical intervention to prevent long-term tissue damage caused by chronic hypoxemia and to avoid potentially fatal complications.

The diagnostic approach to PPHN is multifaceted, involving a combination of non-invasive and invasive methods tailored to assess the severity and implications of the condition effectively. Pulse oximetry and arterial blood gas analysis are crucial for monitoring oxygen saturation and assessing gas exchange capabilities [6].

The use of the oxygenation index (OI) complements these measures by quantifying the degree of respiratory support required to achieve adequate oxygenation. It serves as a vital tool for determining the severity of pulmonary dysfunction and guiding decisions regarding the escalation of care, including the potential need for ECMO when conventional therapies fail to maintain sufficient oxygen levels in the blood [10].

$$\left(\frac{MAP\times {FiO}_{2}}{{PaO}_{2}}\right)\times 100$$

MAP—Mean Airway Pressure; FiO_2_—Fraction of Inspired Oxygen; PaO_2_—Partial Pressure of Arterial Oxygen

The OI value provides information about the severity of hypoxemia and the effectiveness of the respiratory support being provided.

OI < 10: This range suggests mild hypoxemia. It indicates that the current respiratory support is sufficient, and the patient is responding well to the given oxygen and ventilation settings.

OI 10–25: Values within this range represent moderate hypoxemia. It suggests that the patient may require closer monitoring and possibly adjustments in respiratory support to improve oxygenation.

OI > 25: An OI greater than 25 indicates severe hypoxemia and implies a significant risk of pulmonary dysfunction. Patients with an OI above 25 often require intensive interventions, potentially including the use of ECMO to ensure adequate oxygenation and to support lung function.

Chest radiography complements these assessments by visualizing lung fields for associated pathologies such as pulmonary hypoplasia or other lung diseases impacting pulmonary pressure [12].

Although PPHN can be diagnosed clinically in most cases, echocardiography remains the investigation of choice for confirmation of diagnosis, ruling out critical congenital heart diseases and establishing severity of acute pulmonary hypertension [13]. The following structural heart defects may clinically mimic PPHN: TAPVC, transposition of great arteries (TGA), pulmonary atresia with or without ventricular septal defect (VSD), severe Fallot’s tetralogy, tricuspid atresia, unguarded tricuspid orifice syndrome, severe Ebstein anomaly, and sometimes even left-sided obstructive heart disease (such as coarctation of aorta, interrupted aortic arch, and hypoplastic left heart disease) [14].

Detailed overview of echocardiographic assessments is provided in Table 1 and various echocardiographic parameters are illustrated in Figure 1, Figure 2, Figure 3, Figure 4, Figure 5, Figure 6, Figure 7, Figure 8, Figure 9 and Figure 10. Chart 1 summarizes a 7-step approach to a comprehensive assessment of pulmonary hypertension.

**Figure 4:**
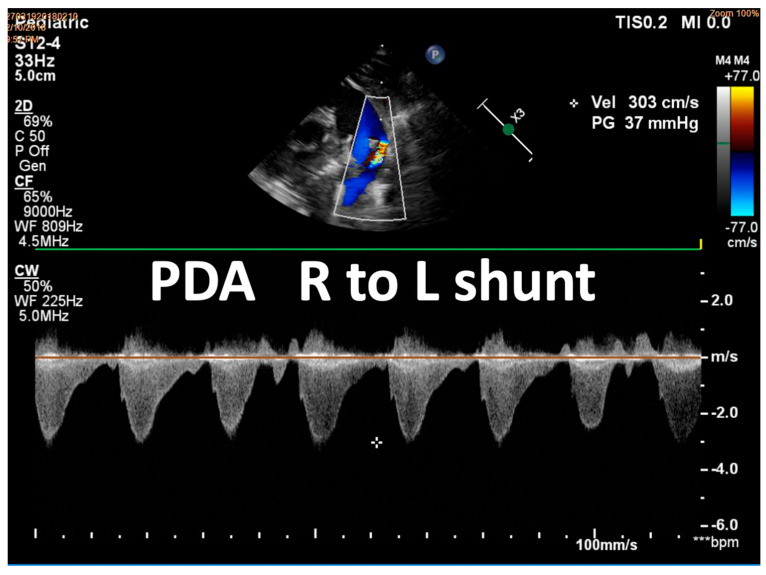
Image showing PDA R to L shunt.

**Figure 5:**
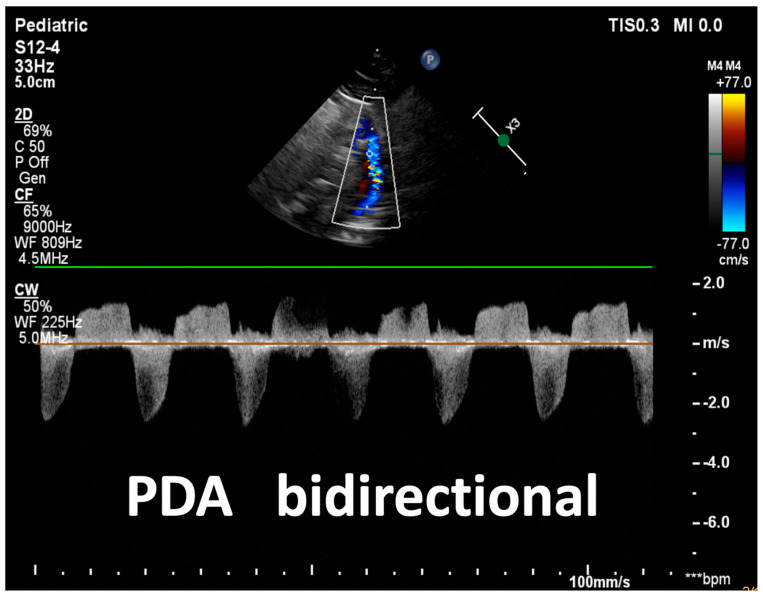
Image showing PDA bidirectional.

**Figure 6:**
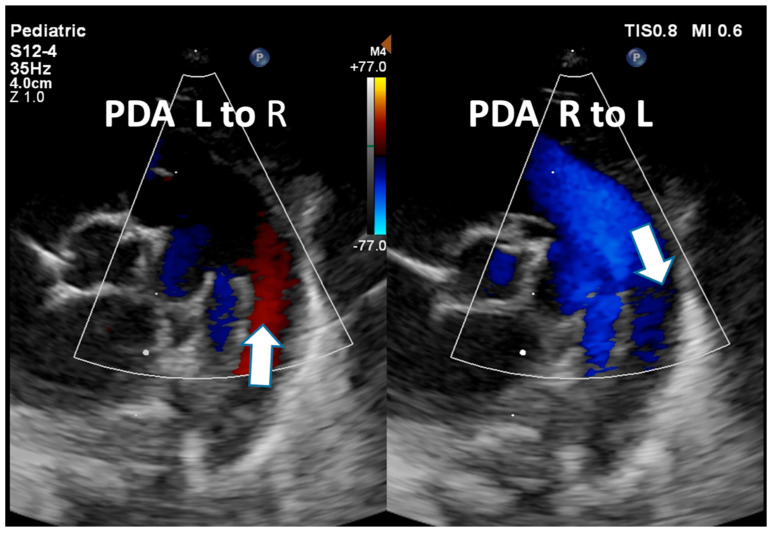
Image showing flow across PDA: red—L to R shunt; blue—R to L shunt.

**Figure 7:**
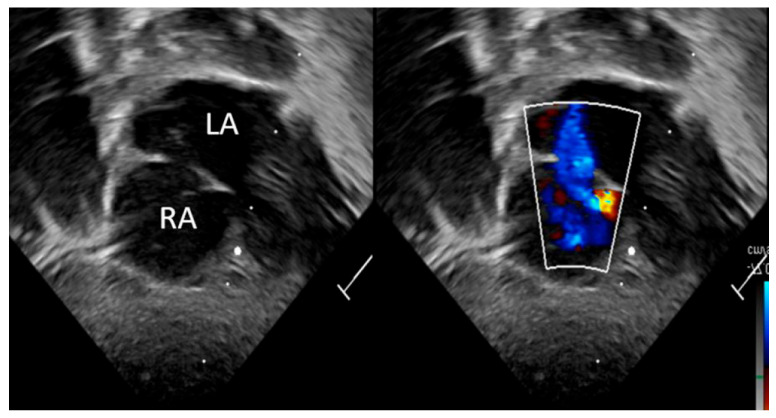
Image showing R to L shunt across PFO.

Figure 4, Figure 5, Figure 6 and Figure 7. Assessment of shunts across PDA and PFO (R to L across PFO demonstrated in figure).

**Figure 9:**
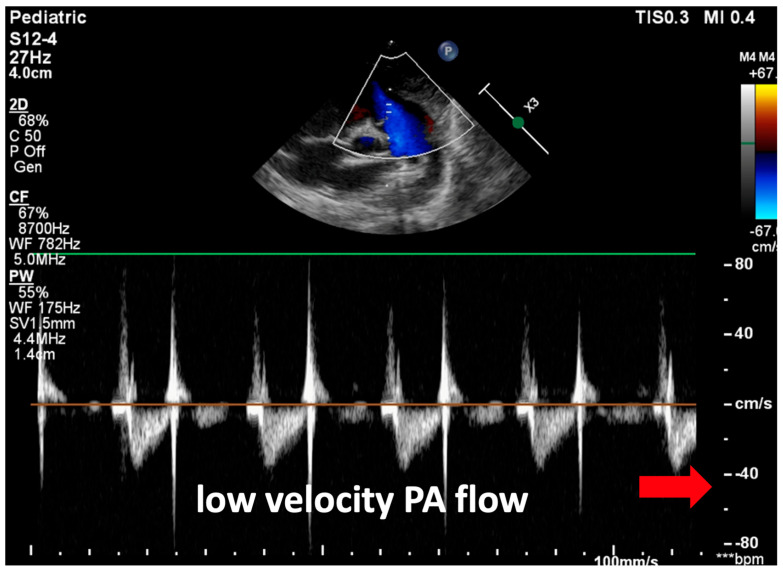
Image showing low velocity PA flow.

**Figure 10:**
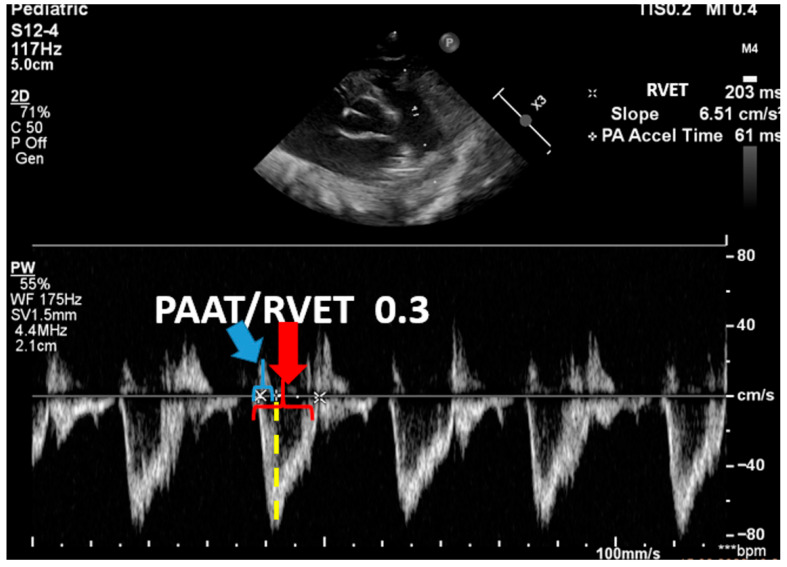
Image showing PAAT and PAAT/RVET measurement.

Figure 9 and Figure 10. Evaluation of PA Dopplers—shape, PAAT, and PAAT/RVET.

For cases where non-invasive methods yield inconclusive results or when precise hemodynamic measurements are required to guide management, cardiac catheterization is employed. This invasive technique allows for direct measurement of pulmonary artery pressures and assessment of vascular reactivity to vasodilators, providing definitive diagnostic data [2,15].

Additionally, the use of biomarkers like B-type Natriuretic Peptide (BNP) or N-terminal pro-B-type Natriuretic Peptide (NT-proBNP) plays a role in evaluating myocardial strain and cardiac stress due to high pulmonary pressures [5].

B-type Natriuretic Peptide (BNP) and its inactive cleavage product N-terminal pro-B-type Natriuretic Peptide (NT-proBNP) are released from ventricular myocardium in response to pressure or volume overload and correlate with right ventricular strain in PPHN. BNP has a short plasma half-life (~20 min), while NT-proBNP is more stable (~60–120 min), making it easier to measure reproducibly. Elevated BNP/NT-proBNP values have been consistently observed in neonates with PPHN compared to infants with other causes of hypoxemic respiratory failure. Suggested thresholds vary between studies, but BNP values > 850 pg/mL have been shown to predict the need for inhaled nitric oxide with high sensitivity [16]. However, absolute cut-offs are not standardized and can overlap with other conditions such as sepsis, perinatal asphyxia, or prematurity.

The main utility of BNP/NT-proBNP in PPHN is serial monitoring: trends in concentration may help assess disease severity, response to pulmonary vasodilator therapy, and predict rebound pulmonary hypertension during weaning. Their role is supportive rather than diagnostic, and values must be interpreted in conjunction with echocardiography and clinical findings.

Emerging biomarkers under investigation include cardiac troponins (reflecting myocardial injury), endothelin-1 (linked to pulmonary vascular tone), and circulating microRNAs, which may provide additional insight into disease mechanisms and prognosis [17].

Integrating findings from these diverse diagnostic tools is crucial for forming a comprehensive understanding of an infant’s cardiopulmonary status.

This holistic assessment aids in customizing therapeutic strategies, determining appropriate intervention timings, and longitudinally monitoring treatment efficacy. Screening protocols recommend specific timing for echocardiographic evaluation, especially in infants predisposed to PPHN due to factors like hypoxia or unresolved respiratory distress, ensuring timely detection and management of this critical condition [18,19,20].

## 5. Treatment

Because of its multifactorial etiology and partially unknown pathophysiological mechanisms, treatment of PPHN remains a challenge. The aim of treatment is to reduce pulmonary vascular resistance (PVR) and reverse blood flow through the shunts. Optimal ventilation after birth is the first crucial factor influencing reduction in PVR [21]. Animal models suggest that physiological cord clamping might be beneficial for children at risk of PPHN, but more research is needed in this field [22,23]. An infant with PPHN with severe respiratory failure requires mechanical ventilation. In order to prevent further lung damage, a lung protective strategy should be applied [24]. In cases where the underlying disease is associated with surfactant deficiency, according to the 2022 European Consensus Guidelines on the Management of Respiratory Distress Syndrome, surfactant administration is a key intervention and leads to improved oxygenation and reduced pulmonary vascular resistance [24].

Management steps for PPHN are shown in Table 2 and Table 3.

### 5.1. Inhaled Therapies

Oxygen is a known potent pulmonary vasodilator; however, hyperoxia also leads to an increase in PVR and the formation of free radicals which causes lung damage and thus should be avoided. In a study carried out by Lakshminrusimha et al., the authors recommend maintaining preductal oxygen saturations in low to mid-90s (93–97%) during management of infants with PPHN with PaO_2_ levels between 55 and 80 mmHg [25].

To reverse blood flow through the shunts, systemic blood pressure should be maintained in proper values. Inotropic agents might often be needed, including dopamine, dobutamine, and epinephrine.

Inhaled nitric oxide (iNO) remains the only registered drug in treatment of PPHN. It works by selectively lowering pulmonary pressure without affecting systemic pressure. The initial starting dose is 20 ppm [25,26]. As reported in a review by Cookson and Kinsela, with no response, the trial of increasing to 40 or 80 ppm is acceptable. Since the half-life of iNO is 2–6 s, the response, i.e., a minimum increase of 20 mmHg, should follow after 30 min [27]. Lower starting doses of 5–10 ppm are used in premature babies.

There is inconsistent interpretation of the OI value as an indicator of both iNO and ECMO initiation. Considering the start of iNO, some authors point to the value of 15, others to one of 25; however, in most reports, the final indicator is 20. The traditional ECMO OI starting threshold was 40 for more than 4 h; however, it is reasonable to consider contacting the ECMO center when, despite implemented therapies, OI still remains above 25, in order to prepare earlier for transportation logistics [28].

### 5.2. Other Pulmonary Vasodilator Therapies

*Sildenafil* is a PDE-5 inhibitor and, as such, leads to vasodilation. It is typically used in adult and pediatric patients with pulmonary hypertension and is increasingly being used in neonates. Although intravenous sildenafil seems more feasible in sick infants not receiving any oral feeds, it carries a higher risk of increased hypotension as compared to oral sildenafil, which is equally effective and has a significantly lower risk of hypotension [29,30]. Moreover, IV sildenafil may not be readily available in many hospitals. A recent meta-analysis showed that sildenafil used for treatment of PPHN has the potential to reduce mortality and improve oxygenation in neonates [30]. Sildenafil is commonly used as a second-line agent in iNO non-responsive patients or as a bridge therapy to wean iNO. However, in limited resource settings where iNO is not available, sildenafil is often used as a first-line pulmonary vasodilator in treating pulmonary hypotension in neonates. A promising experimental study has shown that antenatal use of sildenafil might be beneficial in congenital diaphragmatic hernia (CDH). It would be especially beneficial for those patients, as CDH is not responsive to iNO [31].

Prostanoid analogs (such as iloprost, epoprostenol, and treprostinil) act via the prostacyclin (PGI_2_) pathway, stimulating cyclic adenosine monophosphate (cAMP) to induce pulmonary vasodilation and inhibit platelet aggregation. Evidence for their use in neonates with PPHN is limited to small case series and observational studies; no large randomized neonatal trials exist. Inhaled iloprost has been reported to improve oxygenation indices in iNO-non-responsive infants and is generally well tolerated. Delivery logistics are important: inhaled formulations can be administered to both ventilated and spontaneously breathing infants, whereas continuous intravenous prostacyclins (epoprostenol and treprostinil) require central vascular access and careful hemodynamic monitoring. Side effects include systemic hypotension, flushing, and gastrointestinal symptoms. Because of the limited neonatal data and practical challenges, prostanoids are usually considered as rescue therapies in severe, refractory cases of PPHN [32,33].

Milrinone, a phosphodiesterase-3 inhibitor, increases intracellular cyclic adenosine monophosphate (cAMP), leading to pulmonary vasodilation and improved myocardial contractility. When used as an adjunct to inhaled nitric oxide, it may benefit infants with PPHN who have poor response to iNO alone. Small neonatal case series and observational studies suggest that milrinone can improve oxygenation indices and echocardiographic measures of ventricular performance. However, the evidence base remains limited, with no large randomized trials. Clinicians should exercise caution as milrinone may cause systemic hypotension, particularly in preload-depleted infants; careful assessment of intravascular volume and consideration of additional inotropic support are recommended. Thus, milrinone may be considered in neonates with PPHN and concomitant ventricular dysfunction or low cardiac output, but its use should be individualized and closely monitored [34,35].

Bosentan is an oral dual endothelin-1 receptor antagonist that reduces pulmonary vascular resistance by blocking both ETA and ETB receptors. Limited neonatal experience, mostly from small cohorts and case series, suggests that bosentan can improve oxygenation and allow reduction in inhaled nitric oxide or other vasodilators. One exploratory randomized trial (FUTURE-4) provided preliminary safety and efficacy data, but robust neonatal-specific evidence is still lacking [36]. Typical dosing used in neonates is 1–2 mg/kg every 12 h, with onset of action usually observed within hours to days. Regular monitoring of liver function tests is essential because of the risk of hepatotoxicity, and hemoglobin should be followed due to occasional anemia. Given the current evidence, bosentan should be considered as an adjunct or rescue therapy in refractory PPHN, but its use remains off-label and best guided by specialist consultation [37].

Recent scoping review concluded that the prevalence of PPHN in low- and middle-income countries (LMICs) is increased as compared to high-income medical systems [38]. However limited access to certain therapies in LMICs (iNO and ECMO) have been addressed in several papers [38,39]. Excluding iNO, PPHN is treated with off-label medications; therefore, still more data and studies are warranted to support advantages of selected medications. According to recent meta-analyses, milrinone and oral sildenafil combination results in the most effective treatment when iNO is not available [26,39].

**Chart 2:**
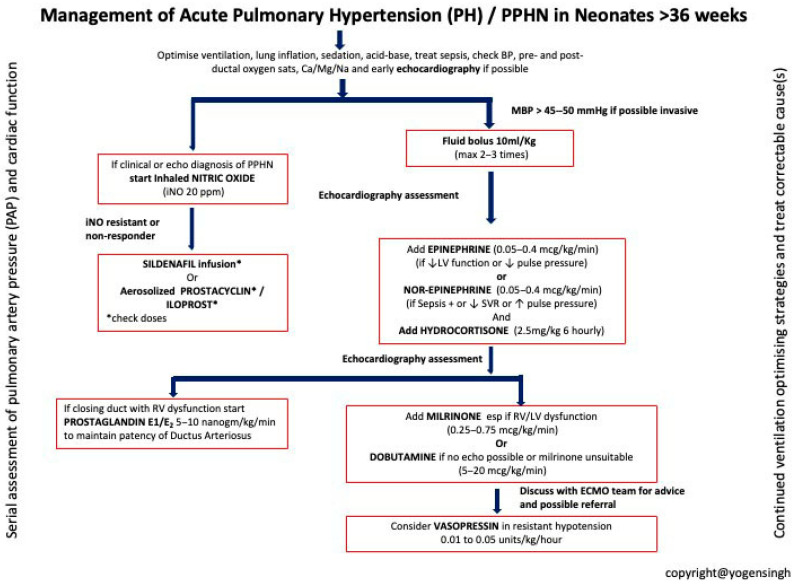
Management of PPHN in centers where iNO is readily available.

**Chart 3:**
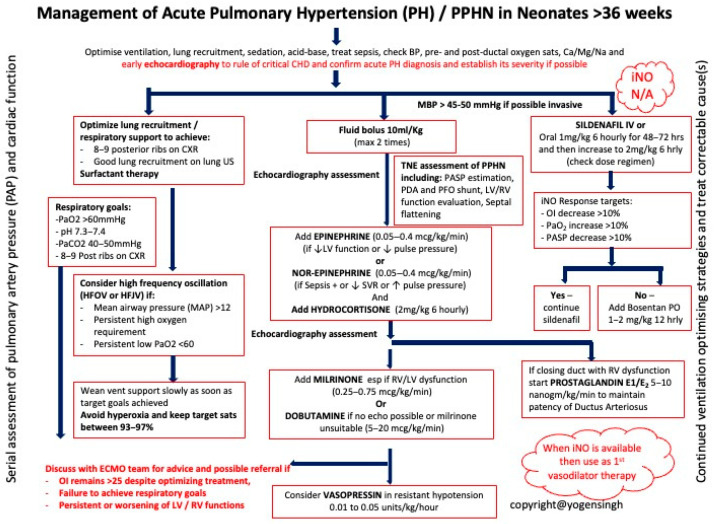
Management of PPHN in limited resource settings where iNO is not readily available.

Hence, we propose an algorithm in this clinical scenario (Chart 2 and Chart 3). Other authors suggest different drug protocols depending on clinical constellation, i.e., normal/low pressure vs. good cardiac function/dysfunction [40]. Specific situations in developing countries regarding thiamine-responsive acute pulmonary hypertension have also been addressed in the literature [41]. Moreover, in resource-limited settings, additional medications, e.g., either inhaled or intravenous magnesium sulfate or nebulized nitroglycerin, have been proposed [42,43]. Recommendations for supportive measures and pharmacotherapy have been addressed in the updated collaborative consensus of the EPPVDN (European Pediatric Pulmonary Vascular Disease Network) [44] and also in comprehensive guidelines pointing out scientific rationale in PPHN management [45] as well as a recent review on treatment [46]. As well as pointing out detailed evidence in procedures and therapeutics, the above mentioned studies and, particularly, meta-analyses and reviews point out the need for further collaboration and planning comparative studies to optimize diagnostic and therapeutic approach to improve the low and moderate quality of the evidence [26,28,38,39].

PPHN is associated with a risk of long-term complications that can affect a child’s development. In total, 10–30% of children who survive PPHN experience some form of neuro-psychomotor developmental disorder later in life [47]. For this reason, multidisciplinary follow-up care after hospital discharge is essential.

A detailed overview of drugs used in the treatment of PPHN is presented in Table 4. Chart 2 summarizes the management of PPHN centers where iNO is readily available, while Chart 3 summarizes the management of PPHN in limited resource settings where iNO is not readily available.

## 6. Conclusions

PPHN is a complex and life-threatening condition requiring early recognition and individualized management. Despite advances in neonatal care, it remains a major cause of neonatal morbidity and mortality. Although it can be diagnosed clinically, echocardiography remains the investigation of choice at the bedside. Accurate diagnosis with the use of echocardiography is needed to understand types of phenotypes, assess severity, target specific intervention and monitor response to intervention.

Inhaled nitric oxide is the mainstay therapy in the western world, but many infants require additional or alternative treatments such as sildenafil, milrinone, or ECMO. Future priorities include developing standardized guidelines, ensuring access to therapies in low-resource settings, and expanding training in neonatal echocardiography. Simultaneously, ongoing exploration of novel pharmacological therapies is essential to expand the therapeutic arsenal and to improve outcomes.

## Figures and Tables

**Figure 2:**
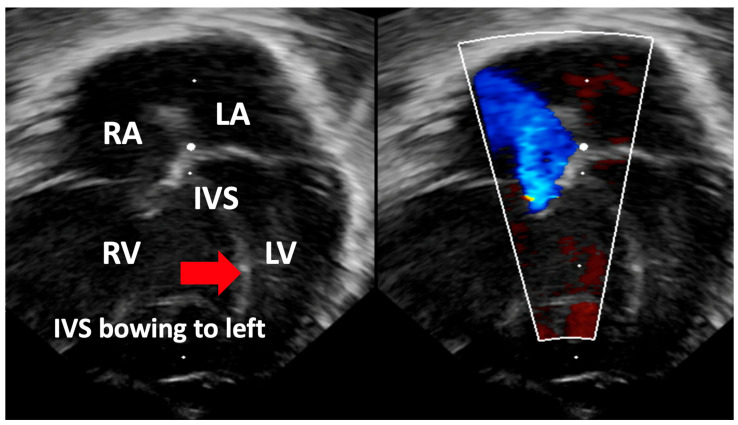
RV dilatation. Image showing bowing IVS to the left because of high pulmonary pressure and tricuspid regurgitation.

**Figure 3:**
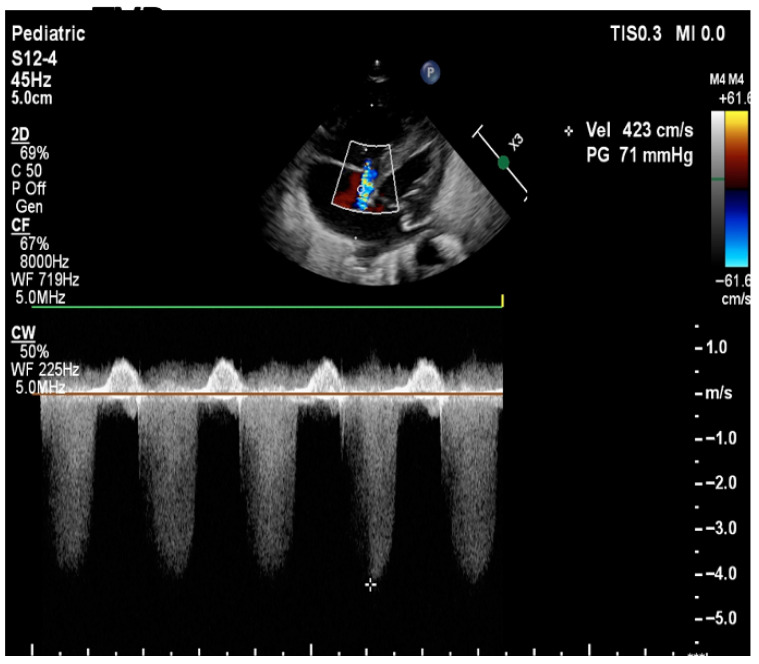
Estimation of pulmonary artery systolic pressure (PASP). Image showing tricuspid regurgitation (TR). Peak velocity of TR estimates pressure gradient between right ventricle and right atrium.

**Figure 8:**
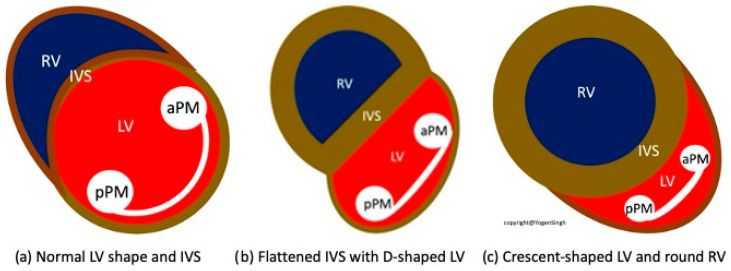
Assessment of inter-ventricular septum and LV shape—(**a**) mild flattening, (**b**) moderate flattening and (**c**) severe flattening of interventricular septum reflecting pulmonary hypertension severity.

**Chart 1:**
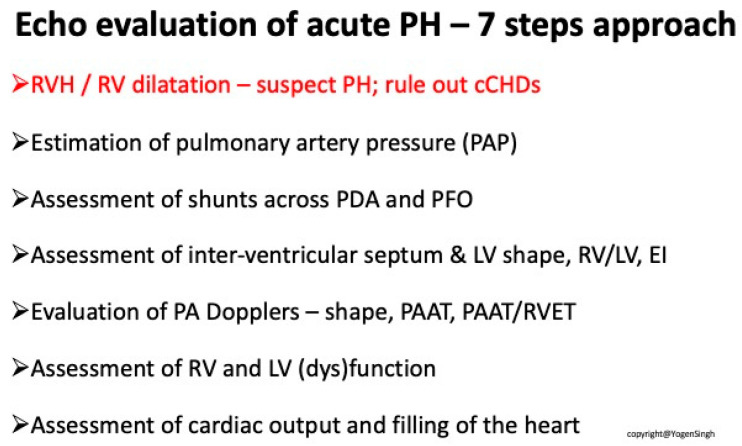
Summary of the 7-step approach to a comprehensive assessment of pulmonary hypertension.

**Table 1:** Overview of echocardiographic assessments for PPHN.

Aspect of Study	Description	Significance
Heart Morphology	Exclusion of structural heart defects (note on Total Anomalous Pulmonary Venous Connection, TAPVC)	Assessment for indications for other treatment such as prostaglandin E
Heart Function	On visual inspection “eyeballing” Evaluation of the size, wall thickness, and contractile function of the right ventricle	Identification of right ventricular enlargement and overload
Interventricular Septal (IVS)	Evaluation of interventricular septal flattening	Helps in estimating severity of PPHN, especially in absence of tricuspid regurgitation or PDA (Figure 4) O-shaped LV suggests normal or mildly increased pressure pulmonary artery pressure D-shaped LV with flattening of IVS indicates pulmonary artery pressure 50–100% of systemic pressure Crescent-shaped LV indicates pulmonary pressure > systemic pressure
Pulmonary Artery Pressure Measurement	Estimation based on the velocity of blood flow through regurgitant flow across the tricuspid valve using the Bernoulli equation	SPAP = 4 (TR Vmax2+ right atrial pressure (RAP) V: the velocity of regurgitation through the tricuspid valve RAP: the estimated right atrial pressure (3–10 mmHg). Pressures in the right ventricle and pulmonary artery are crucial for assessing the degree of pulmonary hypertension TV is present in approximately 60–85% of patients with PPHN. Absence of TR or minimal TR does not rule out PPHN!
Detection of Shunt	Identification and assessment of flow through the patent foramen ovale (PFO) and ductus arteriosus (PDA)	Indicates the presence and nature of pathological flow: left-to-right, bidirectional (often in moderate PPHN), or right-to-left (in very severe PPHN)
Right Ventricle Assessment	On visual inspection Movement of the tricuspid valve annulus, TAPSE (Tricuspid Annular Plane Systolic Excursion) Tei index using Tissue Doppler Imaging	Helps evaluate function of the right ventricle
Left Ventricle Assessment	On visual inspection, fraction shortening, ejection fraction, Tei index using Tissue Doppler Imaging	Helps evaluate function of the left ventricle
Assessment of Cardiac Filling	Inferior vena cava size and collapsibility	Assessment of preload
Advanced ECHO and Hemodynamic Evaluation	RV fractional area change (FAC %) Pulmonary arterial acceleration time (PAAT) and PAAT/RV ejection time ratio Speckle tracking and strain rate Estimation of left and right cardiac output and serial assessment to see the response to therapy	Helps evaluate cardiac function and response to therapy

**Table 2:** Management steps for PPHN.

Step in Management	Actions and Considerations
Initial Assessment and Stabilization	Assess the newborn after birth for breathing, skin color, activity, and oxygenation. Ensure airway patency, provide adequate ventilation, and optimize oxygen concentration.
Diagnostics	Pulse Oximetry: Monitor oxygen saturation. Echocardiography: Assess heart function and exclude structural abnormalities. Arterial Blood Gas Analysis: Evaluate hypoxemia and acid–base balance. Chest X-Ray: Assess lung condition and exclude other causes of respiratory distress.
Intensive Therapy	Optimized Ventilation: Use gentle mechanical ventilation to minimize barotrauma. Inhaled Nitric Oxide (iNO): First-line therapy for PPHN to decrease pulmonary vascular resistance. Surfactant Therapy: Administer if respiratory distress syndrome (RDS) is present. Sedation and Hemodynamic Support: Manage discomfort and maintain hemodynamic stability. Metabolic Correction: Address electrolyte and acid–base imbalances.
Alternative and Escalation Therapies	ECMO (Extracorporeal Membrane Oxygenation): Consider for severe PPHN unresponsive to other treatments. Vasodilator Drugs: Consider sildenafil and other vasodilators to reduce pulmonary artery pressure.

**Table 3:** An approach to phenotype-based diagnosis, clues to diagnosis, and approach to specific therapy.

	PH Type 1 Phenotype—Increased PVR	PH Type 2 Phenotype—Increased BF	PH Type 3 Phenotype—Increased PVR
Diagnosis clues	History of pre-disposing factors such as MAS, perinatal asphyxia, prolonged premature rupture of membranes	History of conditions leading to increased pulmonary blood flow such as persistent patent ductus arteriosus, ASD, VSD, congenital arterio-venous malformation, etc.	History of pre-disposing factors such evolving BPD, especially severe IUGR with BPD, worsening LV dysfunction
	Significant difference between pre- and post-ductal sats difference	No significant difference between pre- and post-ductal sats difference	Difference between pre- and post-ductal sats difference when PDA open but often closed
	Oligemic lung fields on chest X-ray	Plethoric lung fields on chest X-ray	Non-homogenous lung fields on chest X-ray
	Classical signs of pulmonary hypertension on echo (see text)	Volume overloading of right side of heart, specific lesions leading to significant left to right shunt	Signs of pulmonary vein stenosis or severe LV dysfunction on echo
Guide to targeted specific therapy	Optimize ventilation, pulmonary vasodilators, optimize hemodynamic support	Optimize ventilation, diuretics and treatment of specific lesion, optimize hemodynamic support	Optimize ventilation, treatment of specific lesion such pulmonary vein stenosis or improve LV function with lusitropic drugs such as milrinone

**Table 4:** PPHN treatment—drugs, dose, action sites, and indications.

Drug/Dose	Action Mechanism	Side Effects	Indications
First-line therapies			
Inhaled Nitric Oxide (iNO) 5–20 ppm	Activates soluble guanylate cyclase in vascular smooth muscle → selective pulmonary vasodilation	Methemoglobinemia (dose-related), decreased platelet aggregation	Hypoxemic respiratory failure (OI ≥ 25 or PaO_2_ < 100 mmHg on 100% FiO_2_)
Sildenafil IV: Loading 0.4 mg/kg over 3 h; Maintenance 0.067 mg/kg/h Oral: Initial 0.5 mg/kg; Maintenance 1–3 mg/kg q6h	Selective PDE5 inhibitor → increases cGMP, pulmonary vasodilation	Hypotension (more with IV), hypoxemia	Adjunct or alternative to iNO; first-line in limited-resource settings without iNO
Adjunctive therapies			
Milrinone IV: 0.2–1 µg/kg/min (continuous infusion)	PDE3 inhibitor → ↑cAMP → inotropy + vasodilation	Hypotension, arrhythmia, thrombocytopenia [ADD: caution—ensure preload, consider inotropic support]	iNO non-responders with low cardiac output; improves oxygenation and echo indices [ADD: evidence mainly from small neonatal studies]
Bosentan Oral: 1–2 mg/kg q12h	Endothelin-1 receptor antagonist (ETA + ETB)	Hepatotoxicity (↑LFTs), anemia	Adjunct in refractory PPHN [ADD: evidence from small cohorts + one exploratory RCT; onset hours–days; monitor LFTs/hemoglobin]
Rescue/Alternative therapies			
Prostanoids (Iloprost, Epoprostenol, Treprostinil) Iloprost: Inhaled 1–2.5 µg/kg q2–4h Epoprostenol: IV 1–2 ng/kg/min, max 50–80 ng/kg/min Treprostinil: IV/SC as above	Prostacyclin analogs → activate cAMP pathway → vasodilation, antiplatelet effects	Hypotension, flushing, diarrhea [ADD: evidence limited to small neonatal series; delivery logistics differ for ventilated vs. non-ventilated infants]	Rescue therapy in severe, refractory PPHN
Prostaglandin E1 (PGE1) IV: 5–10 ng/kg/min	Maintains ductal patency → allows right-to-left shunting and reduces RV afterload	Apnea, fever, hypotension	[DEL: RV dysfunction with closing DA maintaining ductal patency to offload the RV] [ADD: Selected PPHN cases with RV dysfunction, where ductal patency supports RV offloading and systemic circulation]
